#  Extinction probabilities as a function of temperature for populations of tsetse (*Glossina* spp.)

**DOI:** 10.1371/journal.pntd.0007769

**Published:** 2020-05-07

**Authors:** Elisha B. Are, John W. Hargrove

**Affiliations:** Centre of Excellence in Epidemiological Modelling and Analysis (SACEMA), University of Stellenbosch, Stellenbosch, South Africa; Kenya Agricultural and Livestock Research Organization, KENYA

## Abstract

Significant reductions in populations of tsetse (*Glossina* spp) in parts of Zimbabwe have been attributed to increases in temperature over recent decades. Sustained increases in temperature might lead to local extinctions of tsetse populations. Extinction probabilities for tsetse populations have not so far been estimated as a function of temperature. We develop a time-homogeneous branching process model for situations where tsetse live at different levels of fixed temperature. We derive a probability distribution *p*_*k*_(*T*) for the number of female offspring an adult female tsetse is expected to produce in her lifetime, as a function of the fixed temperature at which she is living. We show that *p*_*k*_(*T*) can be expressed as a geometric series: its generating function is therefore a fractional linear type. We obtain expressions for the extinction probability, reproduction number, time to extinction and growth rates. The results are valid for all tsetse, but detailed effects of temperature will vary between species. No *G. m. morsitans* population can escape extinction if subjected, for extended periods, to temperatures outside the range 16°C–32°C. Extinction probability increases more rapidly as temperatures approach and exceed the upper and lower limits. If the number of females is large enough, the population can still survive even at high temperatures (28°C–31°C). Small decreases or increases in constant temperature in the neighbourhoods of 16°C and 31°C, respectively, can drive tsetse populations to extinction. Further study is needed to estimate extinction probabilities for tsetse populations in field situations where temperatures vary continuously.

## Author summary

Tsetse flies (*Glossina* spp) are the vectors of the African sleeping sickness. We derived expressions for the extinction probability, and mean time to extinction, of closed populations of *G. m. morsitans* experiencing different levels of fixed temperature. Temperature plays a key role in tsetse population dynamics: no population of *G. m. morsitans* can escape extinction at constant temperatures < 16°C or > 32°C. The effect of temperature is more severe if tsetse populations are already depleted. Increasingly high temperatures due to climate change may alter the distribution of tsetse populations in Africa. The continent may witness local extinctions of tsetse populations in some places, and appearances in places hitherto too cold for tsetse.

## Introduction

A bite from a tsetse fly (*Glossina spp*.) infected with a parasite of the genus *Trypanosoma* may cause Human African Trypanosomiasis (HAT), commonly called sleeping sickness in humans, or Animal African Trypanosomiasis (AAT), commonly called nagana in livestock. These tropical diseases have ravaged the African continent for centuries. They pose serious public health and socio-economic problems, especially to rural farmers, who rely on their livestock for daily subsistence, draught power and general economic gain. Sleeping sickness is difficult to diagnose and the treatments are often difficult to administer [1]. Vector control plays an important role in the fight against trypanosomiasis [2], and understanding the population dynamics of the vector is thus crucial for the control or elimination of both sleeping sickness and nagana.

As with all insects, the body temperature of tsetse is largely determined by ambient temperature and all of the flies’ physiological processes are determined by the temperatures that they experience. Tsetse use various behavioural devices to mitigate the effects of extreme ambient temperatures, such that the temperatures they actually experience are less extreme than indicated by temperatures measured in, for instance, a Stevenson screen [3, 4]. Nonetheless, excessively high, or low, temperatures are lethal for them [5, 6]. This is a serious concern for tsetse because, unlike other insects, the genus *Glossina* is characterized by birth rates that do not exceed about 4% per day. It follows that, for mortality rates > 4% per day, mortality exceeds natality, resulting in negative growth rates for tsetse populations. If these negative rates are sustained the tsetse population will be driven to extinction. In theory, therefore, tsetse populations should be relatively easy to control, or even eradicate, using strategies that achieve quite modest increased mortalities. Equally, extinction could result from increases in mortality consequent on environmental changes, including increases in temperature.

As an example of this effect, a study published in 2018 concluded that, over the previous 40 years, there had been a significant increase in temperatures in the Zambezi Valley of Zimbabwe [7]. Specifically, peak temperatures at Rekomitjie Research Station increased by c. 0.9°C from 1975 to 2017, with an increase of c. 2°C at the hottest time of the year (October-November). These increases in temperature were associated with a massive reduction in populations *G. morsitans morsitans* Westwood and, particularly, *G. pallidipes* Austen in the vicinity of Rekomitjie [8]. The research station is in a national park with no agricultural interference that could negatively impact tsetse populations, and there has never been any attempt to control tsetse in the area. That being the case it is reasonable to think that increasing temperature did, indeed, play an important part in mediating declines in tsetse populations. Elsewhere in Zimbabwe, however, other effects were of greater importance than temperature. Thus, there has been, since 1980, rapid expansion of agricultural activity and, particularly in the 1980s and 1990s, vigorous prosecution of tsetse control measures that saw huge reductions in the range of the fly and in the incidence of animal trypanosomiasis [9]. These, largely anthropogenic, effects could be modified by changes in temperature. In areas, such as the Zambezi Valley, where temperatures have historically been at the hot end of the spectrum for tsetse, further increases could contribute to the kinds of population collapse seen at Rekomitjie. In parts of the middle and highveld areas, however, where winter temperatures have historically been too low to support tsetse populations, increased temperatures could tip the balance in favor of the fly. Similarly, in other parts of Africa, it is important to consider the impact of increasing temperatures on the possibility of, at least, local extinction of tsetse in some parts of the continent and of increased potential for the growth of tsetse populations in other parts which were previously too cold for the flies.

Two published works have estimated extinction probabilities and time to extinction for tsetse populations, by assuming fixed environmental states throughout the life history of the flies. The first [10], derived a branching process model for tsetse populations, with the assumption that male and female offspring are produced with equal probability. This assumption is not generally true [11], although the results obtained in [10] are consistent with published results on tsetse biology. The second publication [12] provides a sound mathematical foundation for the results obtained in [10], and assumes a situation where male to female sex ratio can vary anywhere in the open interval (0, 1) and shows how extinction probability depends on the male-female sex ratio in tsetse populations.

In both papers, in order to gauge the importance of various determinants of tsetse population growth, extinction probabilities were calculated for numerous arbitrary combinations of mortality and fertility rates. There was no explicit modelling of the effect of temperature on vital rates, nor thus on its effect on extinction probabilities. In this paper, we develop a version of the stochastic branching process model presented in [10] and [12] in which all of the biological process in the tsetse lifecycle are explicitly dependent on temperature.

In a model developed to describe the tsetse population dynamics of *G. m. morsitans* on Antelope Island, Lake Kariba, Zimbabwe, it was found that, when temperature was used as an independent variable in the model, adding other climatic factors did not improve the fit of the model to data [13]. This suggests that temperature is a key climatic driver of tsetse population dynamics, though we do not discount the importance of other factors such as vegetation cover, humidity and, particularly, access to suitable vertebrate host species. In this study we focus on the impact of temperature on extinction probability, time to extinction, reproduction number and growth rates for tsetse populations in situations where other factors are considered invariant and favorable to the survival of tsetse populations. Even under such circumstances, we show that positive population growth occurs only within quite a narrow band of mean temperature. Sustained temperatures either above or below this band can drive tsetse populations to extinction.

Various workers have obtained mathematical expressions for the temperature dependence of such functions as daily survival probabilities for young, and mature, adult females, and pupae, and the inter-larval period and pupal duration [6, 8, 14–17]. Using these functions allows us to estimate extinction probabilities, time to extinction and mean of tsetse population size at different levels of fixed temperatures.

It was also assumed in the earlier papers that mortality in adult flies was independent of the age of the fly. Evidence suggests that mortality rates are actually markedly higher in recently emerged adults than in mature flies [6, 18–20], and that this difference is particularly severe at extremes of high temperatures [5, 6]. To capture this difference in mortality rates, we assume higher mortalities for adult females that have not ovulated for the first time, compared with the survival probability of all older flies.

We note at the outset that the temperature relations referred to above generally apply to results from studies on *G. m. morsitans* Westwood. The mode of reproduction is identical in all species of tsetse and it appears that, at temperatures in the region of 25°C, there are only minor variations in the rates at which females produce offspring, and at which the offspring develop [11]. Given that all tsetse are poikilotherms, we may also be sure that all rates will be a function of temperature. Nonetheless, the pioneering work of Phelps [5, 15, 21, 22], who measured the detailed effects of temperature on development, and survival, rates of *G. m. morsitans* pupae, have not been carried out in such detail for any other species of tsetse. Similarly, whereas the effects of fly age and environmental temperature on adult mortality have been carefully estimated for this species [18, 20] there is little information available on such detailed relationships for most other species.

We may be confident that the mathematical functions presented here will be applicable, in general, to all tsetse, whether savannah, forest or riverine species, and indicate the general trends in extinction probabilities to be expected in these species with changing temperatures. The functions will, however, only produce meaningful and accurate results if appropriate values can be made available for all of the input parameters for individual species. At the moment this is approximately true only for *G. m. morsitans*.

## Materials and methods

We follow the general approach described earlier [10, 12] but the extinction probability is now obtained with fewer mathematical steps, and in a more compact form. We use the solution to obtain numerical results for extinction probabilities, times to extinction and growth rates for tsetse populations living at various fixed temperatures. We achieve this by using existing functions in the literature relating tsetse fly lifecycle parameters to temperature. We modify published versions of the model [10, 12] by separating the adult life stage of tsetse fly into immature and mature classes, allowing us to assess differential impacts of temperature in the two stages.

### Tsetse life history

The following provides a brief description of the life cycle; fuller accounts are provided in [23]. Unlike most other insects, tsetse have a very low birth rate: they do not deposit eggs, instead producing a single larva every 7–12 days [16, 20]. The larva buries itself in the ground and immediately pupates, staying underground as a pupa for between 30–50 days [21], emerging thereafter as an adult with the linear dimensions of a mature adult, but with poorly developed flight musculature, and low levels of fat. Both sexes of tsetse feed only on blood, and the first 2-3 blood-meals are used to build flight muscle and fat reserves, before the mature female can embark on the production of larvae of her own. All of these processes are temperature dependent. The pupal phase in *G. m. morsitans* increases from 20 days at 32°C to 100 days at 16°C (28 days at 25°C) [22], though few adults emerge at the extremes of temperature. Blood-meals are taken every 2–5 days, again depending on temperature. The time between adult female emergence and first ovulation is only weakly dependent on temperature and is assumed here to take a constant value of 8 days in the field at Rekomitjie [23]. Thereafter, the period between the production of successive pupae increases from about 7 days at 32°C to 12 days at 20°C (9 days at 25°C) [24].

### Model

The model development and assumptions are similar to those in [12], differing only in the way that mortality and fertility rates are used in the models. In the earlier works, these rates were simply set at constant values. In the present study we use the fact that the rates are almost all known functions of the temperature experienced by the flies. Accordingly, instead of setting these rates at arbitrary values, we instead allow the environmental temperature to take various values, which then dictate the values of the rates of mortality and fertility to be used in the model. In particular, the following rates are all temperature dependent; pupal duration and inter-larval period, and the daily survival probabilities for female pupae, adult females that are immature (defined as not having yet ovulated for the first time), and mature adult females. We do not explicitly model the growth of the male part of the population, instead we assume that there are always sufficient numbers of males present to ensure that all females are inseminated.

### Model assumptions

In what follows all parameters with subscript *T* are temperature dependent.

1. An immature adult female tsetse fly [6] survives the *ν* days until it ovulates for the first time with probability $\Omega_{T}=e^{-\nu \omega_{T}}$, where *ω*_*T*_ is the daily mortality rate for immature adult females.
2. A mature female tsetse survives with probability $\lambda_{T}=e^{-\psi_{T}}$ per day, where *ψ*_*T*_, is the daily mortality rate for mature females.
3. Once a female has ovulated for the first time, she deposits a single larva every *τ*_*T*_ days.
4. A deposited larva is female with probability *β*.
5. The larva burrows rapidly into the soft substrate where it has been deposited and pupates [8]. The pupa survives with probability $\phi_{T}=e^{-\chi_{T}}$ per day, where *χ*_*T*_ is the daily mortality rate for female pupae.
6. At the end of the pupal period of *σ*_*T*_ days, an immature adult fly emerges from the puparium.
7. The immature female fly is inseminated by a fertile male tsetse after *ν* days, with probability *ϵ*.

The probability that a female tsetse produces *k* surviving female offspring is obtained as:

$$p_{k}\left(T\right)=\frac{\epsilon \Omega_{T}^{\nu }\lambda_{T}^{k\tau_{T}}\left(1-\lambda_{T}^{\tau_{T}}\right)\beta^{k}\varphi_{T}^{k\sigma_{T}}}{{\left(1-\beta \lambda_{T}^{\tau_{T}}\left(\frac{1}{\beta }-\varphi_{T}^{\sigma_{T}}\right)\right)}^{k+1}},k>0.$$ (1)

The proof of Eq (1) can be easily adapted from the proof of Eq (7) in the supplementary material of [12]. The difference here is simply that the probability is a function of temperature. We assume that *T* is time invariant. Our interest is to estimate extinction probabilities for tsetse living at fixed temperatures.

Eq (1) can be used to derive a function for the extinction using the procedures described in earlier publications [10, 12]. A simpler derivation, using the work of Harris [25], is given here. Suppose *p*_*k*_(*T*) follows a geometric series for all *T*, then:

$$p_{k}\left(T\right)=b_{T}c_{T}^{k-1},$$ (2)

where *b*_*T*_, *c*_*T*_ > 0.

For Eq (1) we then have:

$$b_{T}=\frac{\epsilon \Omega_{T}^{\nu }\lambda_{T}^{\tau_{T}}\left(1-\lambda_{T}^{\tau_{T}}\right)\beta \varphi_{T}^{\sigma_{T}}}{{\left(1-\beta \lambda_{T}^{\tau_{T}}\left(\frac{1}{\beta }-\varphi_{T}^{\sigma_{T}}\right)\right)}^{2}}$$

and

$$c_{T}=\frac{\lambda_{T}^{\tau_{T}}\beta \varphi_{T}^{\sigma_{T}}}{\left(1-\beta \lambda_{T}^{\tau_{T}}\left(\frac{1}{\beta }-\varphi_{T}^{\sigma_{T}}\right)\right)}$$

Inserting *b*_*T*_ and *c*_*T*_ into Eq (2), yields

$$p_{k}\left(T\right)=\frac{\epsilon \Omega_{T}^{\nu }\lambda_{T}^{\tau_{T}}\left(1-\lambda_{T}^{\tau_{T}}\right)\beta \varphi_{T}^{\sigma_{T}}}{{\left(1-\beta \lambda_{T}^{\tau_{T}}\left(\frac{1}{\beta }-\varphi_{T}^{\sigma_{T}}\right)\right)}^{2}}{\left(\frac{\lambda_{T}^{\tau_{T}}\beta \varphi_{T}^{\sigma_{T}}}{\left(1-\beta \lambda_{T}^{\tau_{T}}\left(\frac{1}{\beta }-\varphi_{T}^{\sigma_{T}}\right)\right)}\right)}^{k-1},k\geq 1$$ (3)

It follows (from [25], page 9) that the generating function *f*_*T*_(*s*), for *p*_*k*_(*T*) is a fractional linear function, and can be expressed as:

$$f_{T}\left(s\right)=1-\frac{b_{T}}{1-c_{T}}+\frac{b_{T}s}{1-c_{T}s},0\leq s\leq 1.$$ (4)

### Mean of female tsetse population at generation n

Substituting for *b*_*T*_ and *c*_*T*_ in Eq (4) and taking the first derivative w.r.t *s*, at *s* = 1.

$$\mu_{T}=f_{T}^{\prime }\left(1\right)=\frac{\epsilon \Omega_{T}^{\nu }\lambda_{T}^{\tau_{T}}\beta \varphi_{T}^{\sigma_{T}}}{\left(1-\lambda_{T}^{\tau_{T}}\right)}$$ (5)

Eq (5) is the reproduction number for a female tsetse population. For a population of tsetse living at temperature *T*°C, the expected number of female tsetse in the population at generation *n* is denoted by

$$M\left(n\right)=\mu_{T}^{n}.$$

#### Remark 1

When *μ*_*T*_ > 1, the branching process is said to be supercritical with extinction probability *q*_*T*_ < 1. If *μ*_*T*_ < 1, the branching process is subcritical, which implies, in practice, that each female tsetse produces less than one surviving female offspring on average. Extinction is then certain: i.e., the probability *q*_*T*_ = 1. The process is called critical if *μ*_*T*_ = 1, and extinction probability is again certain, *q*_*T*_ = 1 (see [26] page 36). In other words, for any tsetse population to avoid inevitable extinction, each female fly must produce more than one surviving female offspring in her lifetime.

### Extinction probability *q*_*T*_

The extinction probability is obtained by solving for the fixed points of Eq (4), that is, we find *s* such that *f*_*T*_(*s*) = *s*. We therefore need to solve:

$$1-\frac{b_{T}}{1-c_{T}}+\frac{sb_{T}}{1-sc_{T}}=s$$ (6)

Substituting for *b*_*T*_ and *c*_*T*_ in Eq (6) and solving for *s*, the extinction probability *s* = *q*_*T*_ is the smaller nonnegative root of Eq (6).

$$q_{T}=\frac{1-\lambda_{T}^{\tau_{T}}\left(1-\beta \varphi_{T}^{\sigma_{T}}\left(1-\epsilon \Omega_{T}^{\nu }\right)\right)}{\beta \lambda_{T}^{\tau_{T}}\varphi_{T}^{\sigma_{T}}},$$ (7)

where $\beta \lambda_{T}^{\tau_{T}}\phi_{T}^{\sigma_{T}}\neq 0$.

#### Remark 2

Suppose $\beta \lambda_{T}^{\tau_{T}}\phi_{T}^{\sigma_{T}}<1-\lambda_{T}^{\tau_{T}}\left(1-\beta \phi_{T}^{\sigma_{T}}\left(1-\epsilon \Omega_{T}^{\nu }\right)\right)$, then $\epsilon \Omega_{T}^{\nu }\lambda_{T}^{\tau_{T}}\beta \phi_{T}^{\sigma_{T}}+\lambda_{T}^{\tau_{T}}<1$, which implies that *μ*_*T*_ < 1 (5). Therefore, whenever the denominator of Eq (7) is less than the numerator, extinction probability *q*_*T*_ = 1. Hence, for all biologically meaningful parameter ranges, *q*_*T*_ is always in [0, 1]. See **Remark 1**. Furthermore, when the initial population consists of a single female fly, the extinction probability is given by Eq (7). If the initial population is made up of *N* female flies, the extinction probability is given by (*q*_*T*_)^*N*^ [12].

### Expected time to extinction for a population of tsetse

An expression for the expected time for a population of female tsetse to become extinct is presented in [10], as:

$$E\left(K\right)=\sum_{n=0}^{\infty }\left(1-{\left(q_{n}\left(T\right)\right)}^{N}\right),$$ (8)

where

$$f_{T}\left(q_{n-1}\left(T\right)\right)=\sum_{k=0}^{\infty }p_{k}\left(T\right){\left(q_{n-1}\left(T\right)\right)}^{k}=q_{n}\left(T\right).$$

*K* is the time to extinction and *N* is the number of female tsetse flies in the initial population. To estimate the mean time to extinction of a population consisting of *N* flies, it suffices to calculate *q*_*n*_(*T*) iteratively, and raise each value to the power *N*, to obtain *E*(*k*) as given in Eq (8). Note that, since females produce both male and female offspring, extinction of the female population obviously guarantees the extinction of the whole population.

### Tsetse mortality rates as a function of temperature

**Fig 1:**
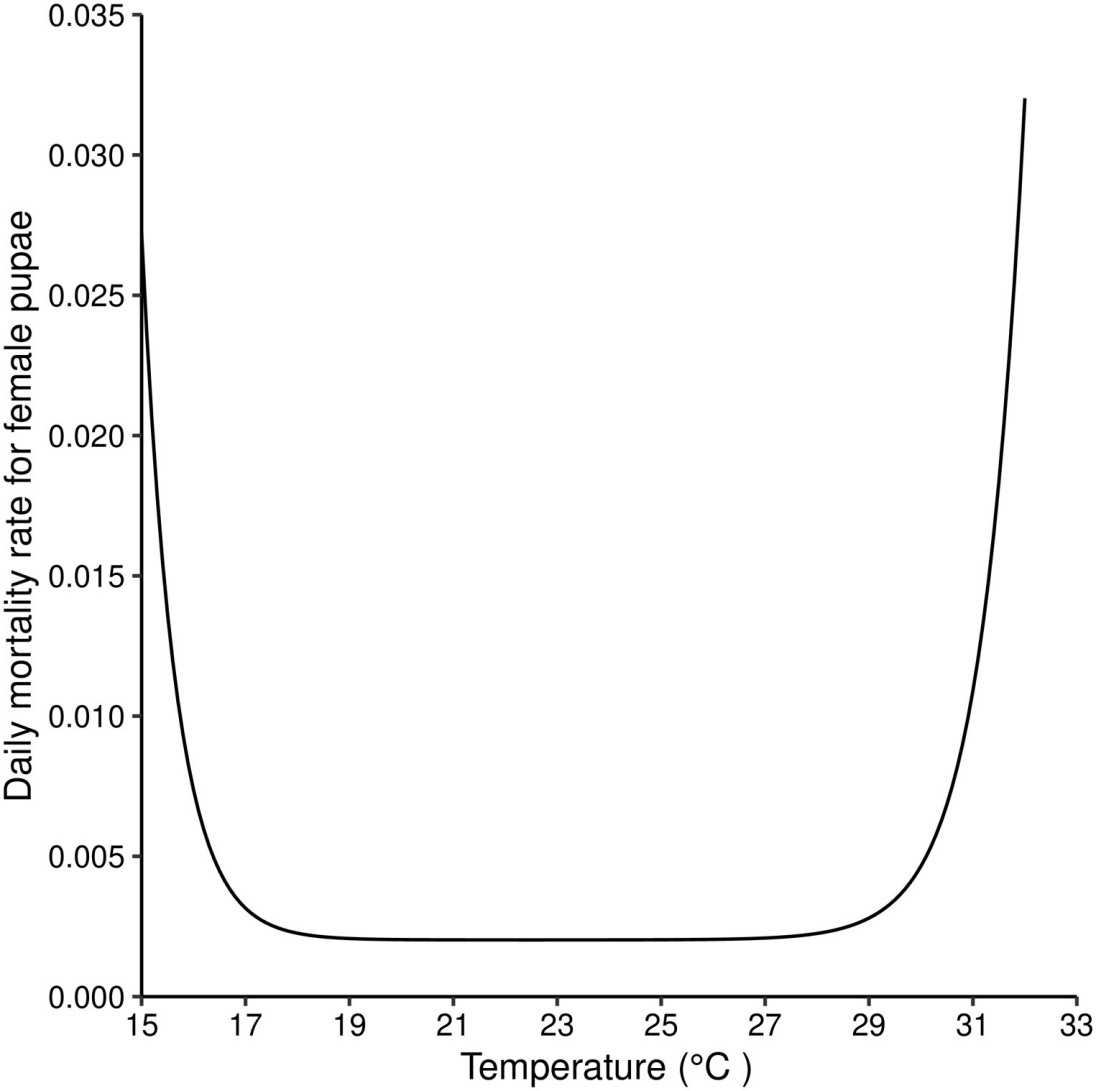
Daily mortality rates for female *G. m. morsitans* pupae for temperatures in the range (15°C–33°C). Eq (9) plotted for different values of temperature.

The relationship between temperature and the instantaneous daily mortality rate of pupae is modelled as a the sum of two exponentials (Fig 1) [14].

$$\chi_{T}=b_{1}+b_{2}e^{-b_{3}\left(T-16\right)}+b_{4}e^{b_{5}\left(T-32\right)}$$ (9)

**Fig 2:**
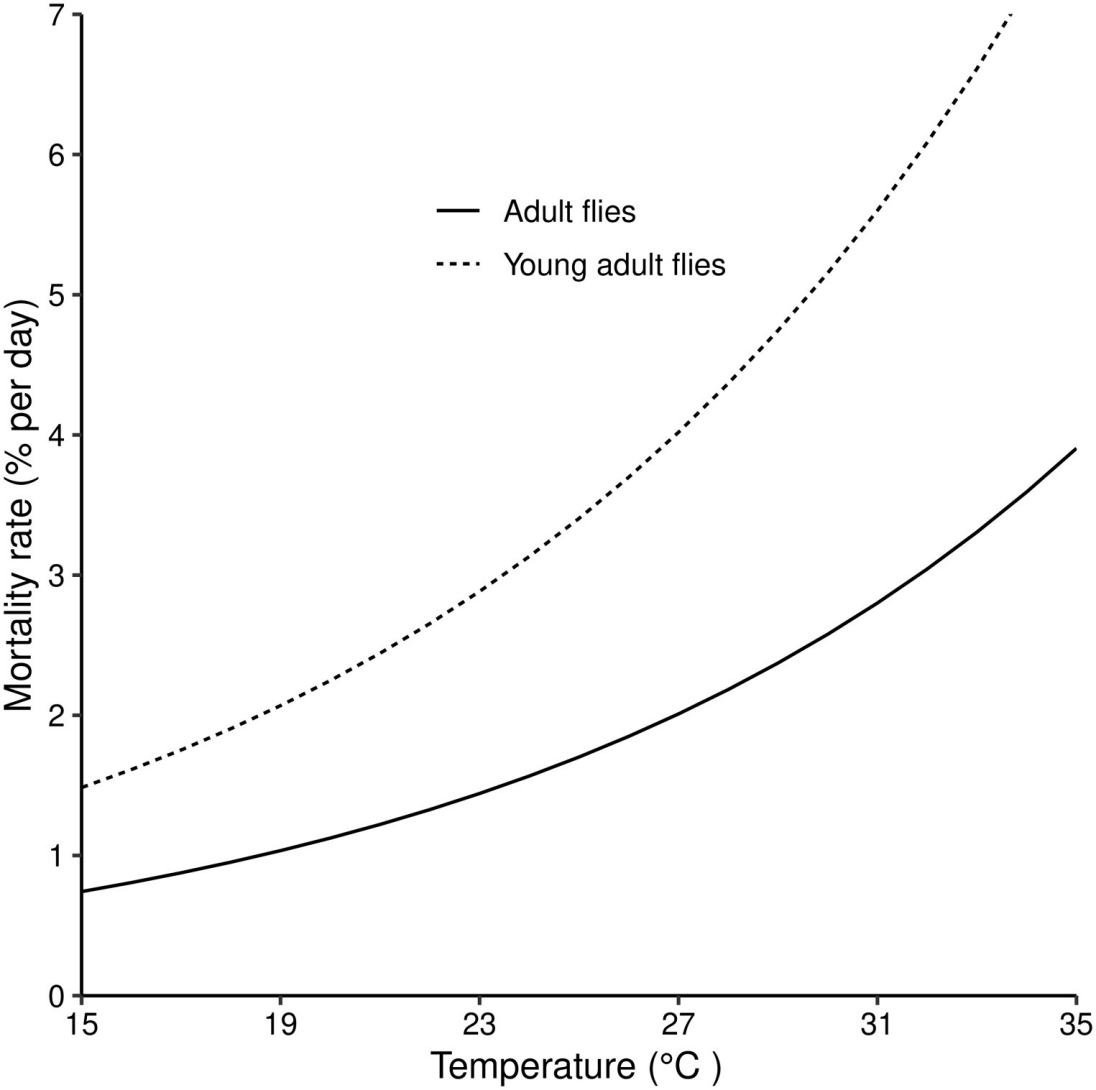
Daily mortality rates for young adult and mature adult female *G. m. morsitans* for temperatures ranging from 15°C–35°C. Eqs (10) and (11) plotted for different temperatures.

Daily mortality rates of young and mature adult female *G. m. morsitans* increase exponentially with temperature (Fig 2). The defining equations, (10) and (11), take the same form, differing only in the parameter values (Table 1), due to the higher mortality of young flies, particularly at high temperatures.

$$\omega_{T}=\frac{e^{-b_{6}+b_{7}T}}{100}$$ (10)

$$\psi_{T}=\frac{e^{-b_{8}+b_{9}T}}{100}$$ (11)

**Table 1:** Summary of model parameter values for *G. m. morsitans* and sources: Parameter values are generally sourced from the literature.

Parameter	Description	Value & Source
	Daily female pupal mortality (*χ*_*T*_)	[14]
*b*_1_	Eq (9)	0.00202
*b*_2_		0.00534
*b*_3_		1.552
*b*_4_		0.03
*b*_5_		1.271
	Daily mortality rate for young flies (*ω*_*T*_)	[17]
*b*_6_	Eq (10)	-0.85
*b*_7_		0.083
	Daily mortality rate for mature flies (*ψ*_*T*_)	[17]
*b*_8_	Eq (11)	-1.60
*b*_9_		0.083
	Pupal duration (*σ*_*T*_) (days)	[16]
*c*_1_	Eq (12)	17.94
*c*_2_		82.3
*c*_3_		-0.253
	Inter-larval period (*τ*_*T*_) (days)	[17]
*d*_1_		0.1046
*d*_2_	Eq (13)	0.0052
*β*	Probability deposited pupa is female *β*	0.5 [12]
*ν*	Time (days) from adult emergence to first ovulation	8.0 [12]
*ϵ*	Probabilty of insemination by a fertile male	1.0 [12]

### Development rates as a function of temperature

Pupal duration is modelled as increasing exponentially with decreasing temperature [14, 22, 27, 28].

$$\sigma_{T}=c_{1}+c_{2}e^{c_{3}\left(T-16\right)}$$ (12)

The relationship between larviposition rate and temperature was modelled using the results from a mark and release experiment conducted at Rekomitjie on *G. m. morsitans* and *G. pallidipes* [16].

$$\tau_{T}=\frac{1}{d_{1}+d_{2}\left(T-24\right)}$$ (13)

In Eqs (9)–(13), *b*_1_, *b*_2_, *b*_3_, *b*_4_, *b*_5_, *b*_6_, *b*_7_, *b*_8_, *b*_9_, *c*_1_, *c*_2_, *c*_3_, *d*_1_ and *d*_2_ are all constants.

Numerical simulations were carried out using R studio (version 1.1.463) [29], by incorporating Eqs (9)–(13) into Eq (7) and taking parameter values from the literature as shown in Table 1.

We note that it has been demonstrated that computing mean daily fertility in adult female tsetse, using the reciprocal of the interlarval period, under-estimates the true fertility by about 10%: it was also demonstrated that the Antelope Island mark-recapture procedure [17] over-estimated mortality among older tsetse [30, 31]. Our development uses the correct procedure for estimating fertility, and we have adjusted the functions relating adult mortality to temperature as suggested in these studies.

## Results

**Fig 3:**
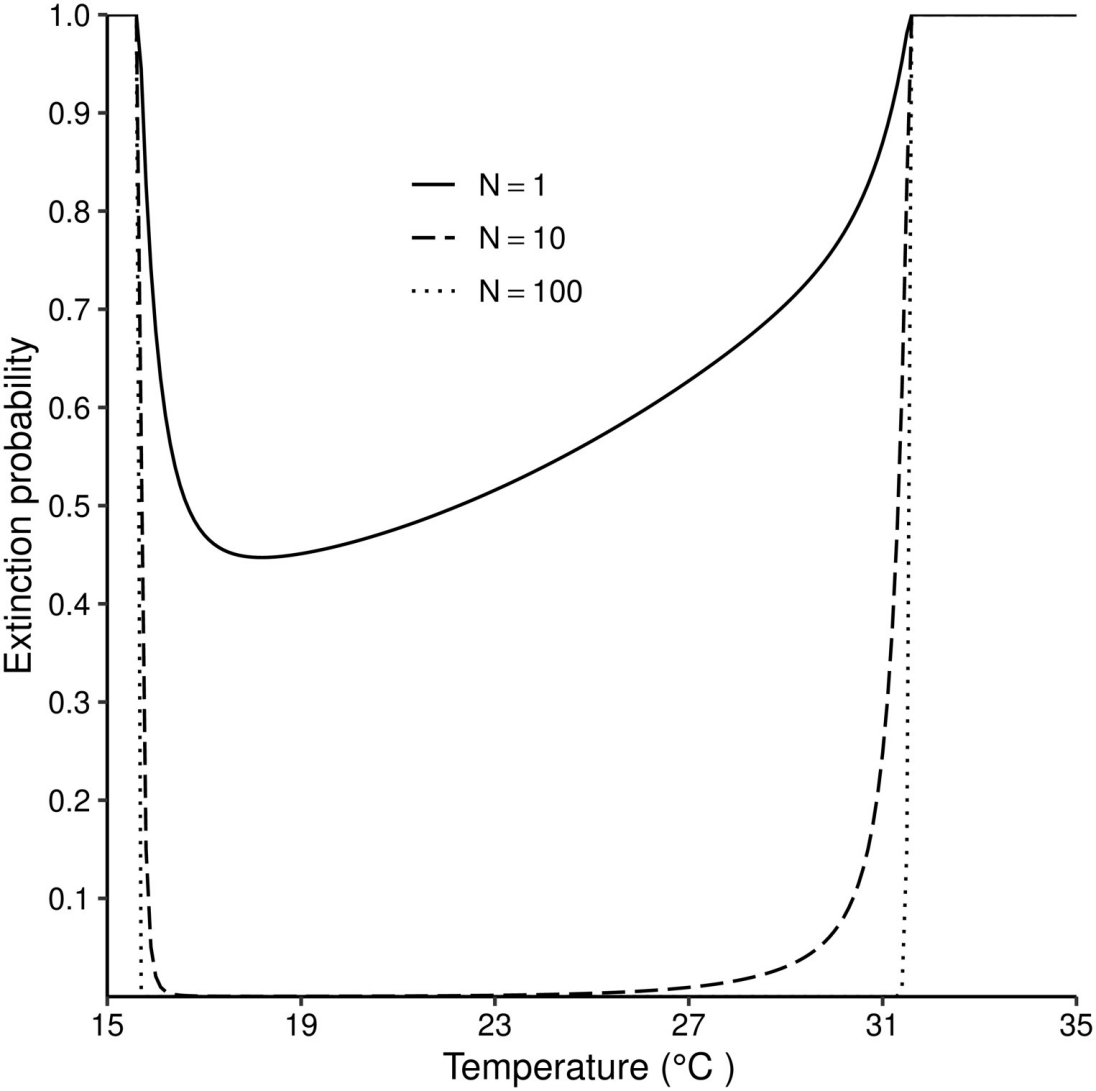
Probability of extinction of a population of *G. m. morsitans* as a function of temperature varying between 15°C and 35°C. Eq (7) solved for different values of temperature and for different numbers of inseminated adult females in the initial population.

**Fig 4:**
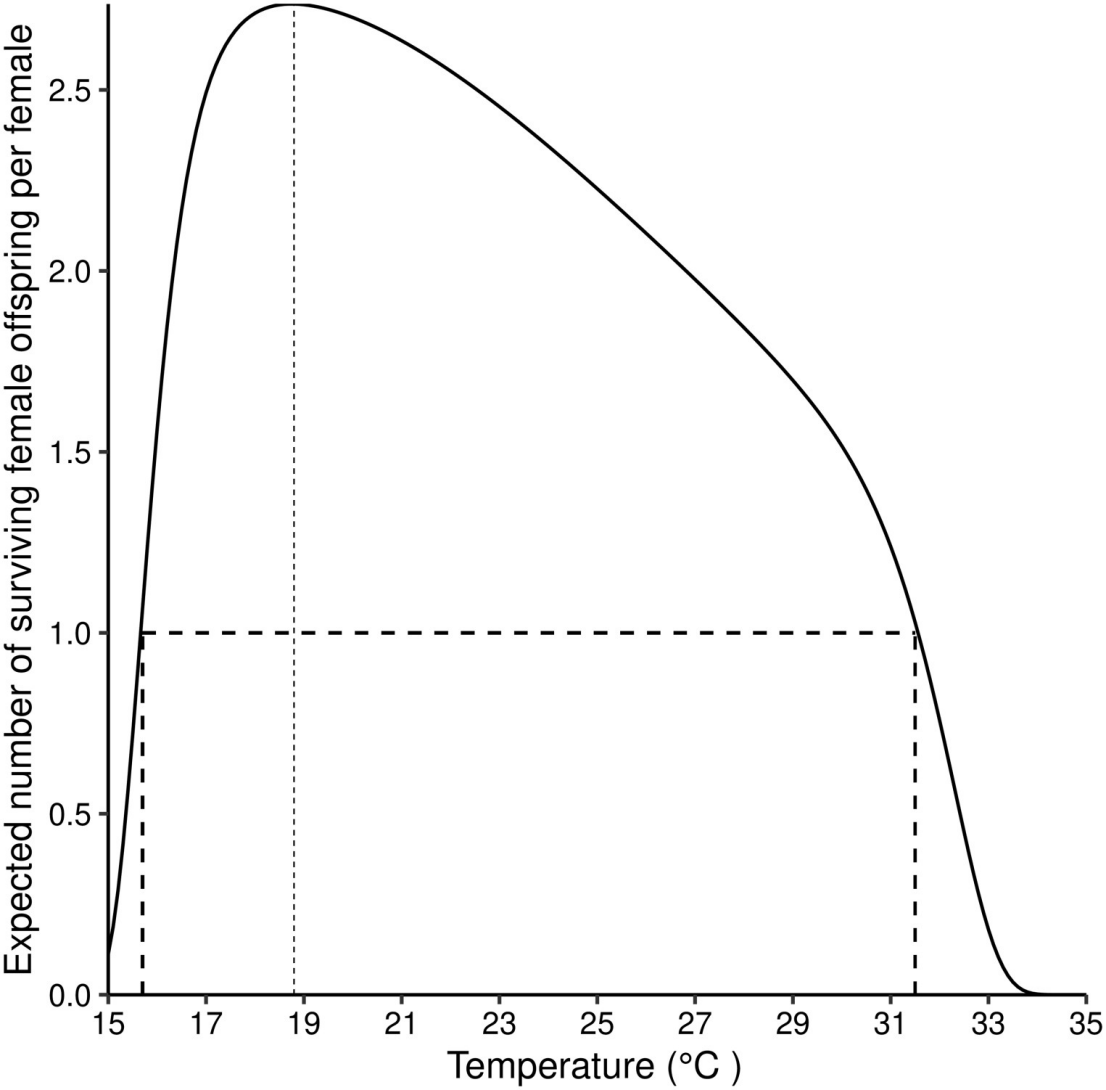
Expected number of surviving female offspring per adult female *G. m. morsitans* for different temperatures (15°C–35°C). Eq (5) solved for different values of temperature. The fine vertical dotted line at about 19°C indicates the temperature at which a female produces the greatest number of surviving daughters. The dashed vertical lines just below 15°C and just above 31°C, respectively, indicate the temperatures below and above which a female produces fewer than one surviving daughter.

**Fig 5:**
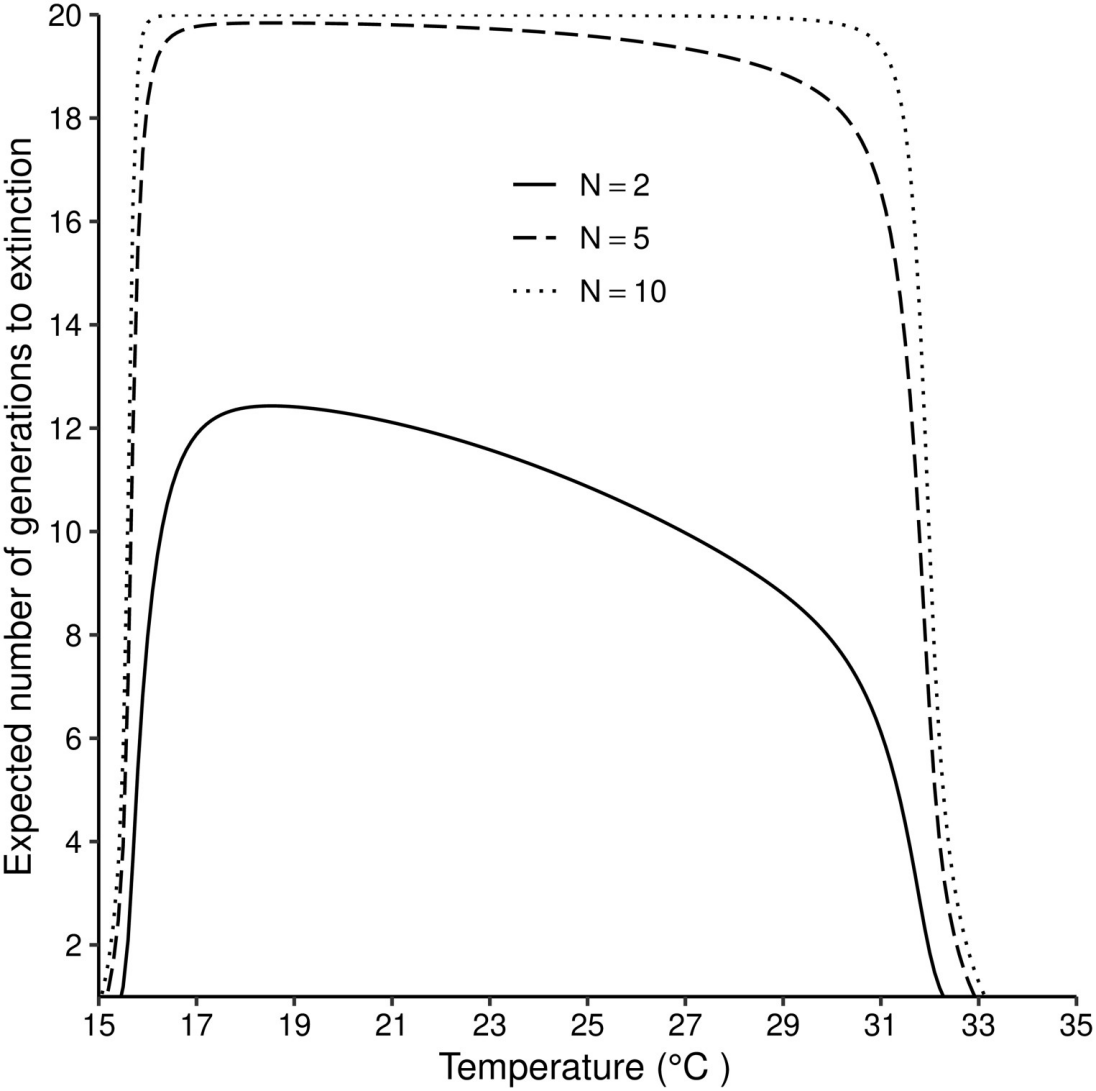
Expected number of generation to extinction of a *G. m. morsitans* population for different number of females in the initial population at different temperatures (15°C–35°C). Eq (8) is solved iteratively up to *n* = 20.

**Fig 6:**
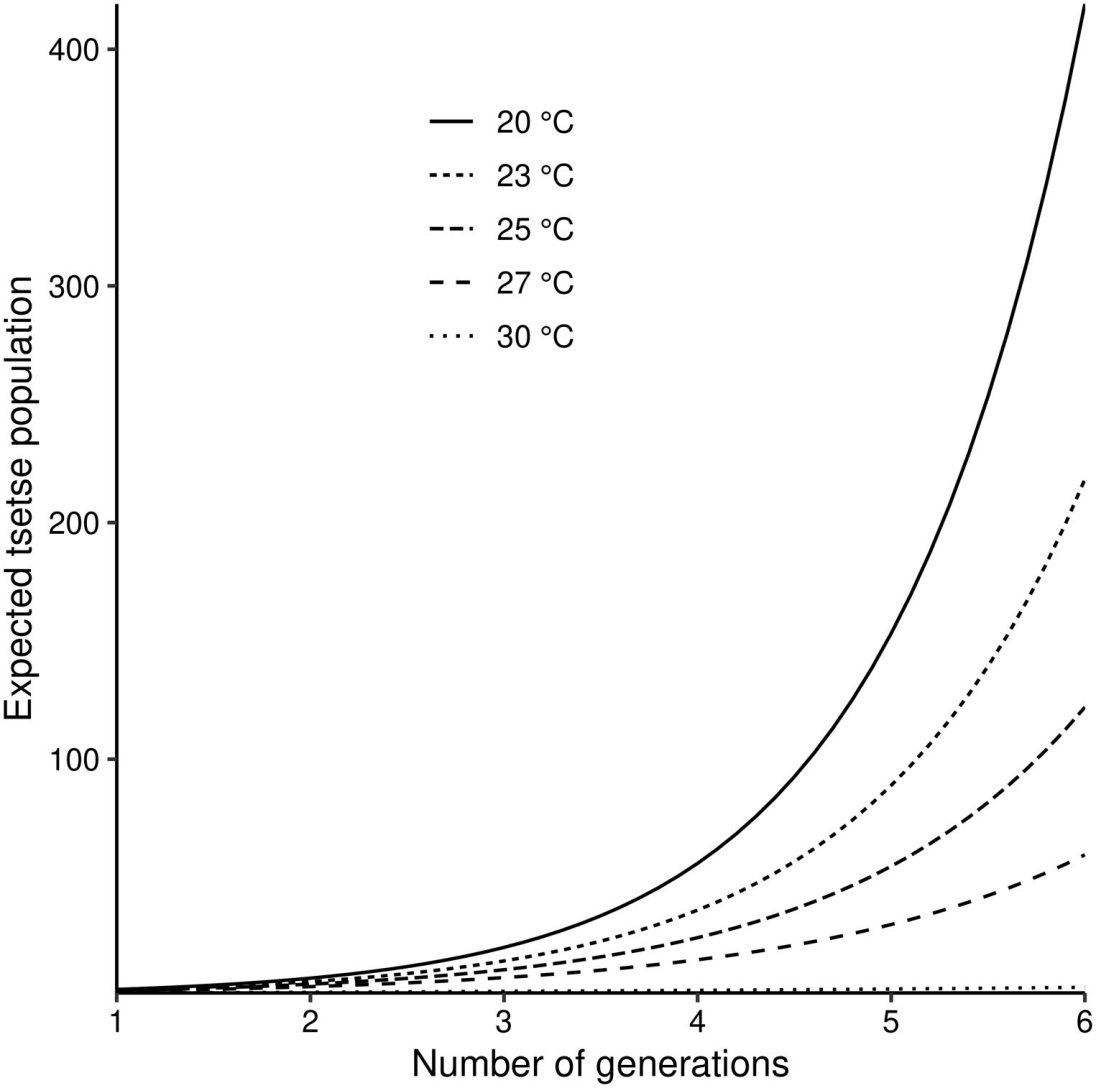
Expected growth in the numbers of adult females in a *G. m. morsitans* population at different temperatures (15°C–35°C). The projections are approximately valid for the early stages of growth, before density dependent processes have noticeable effects.

**Fig 7:**
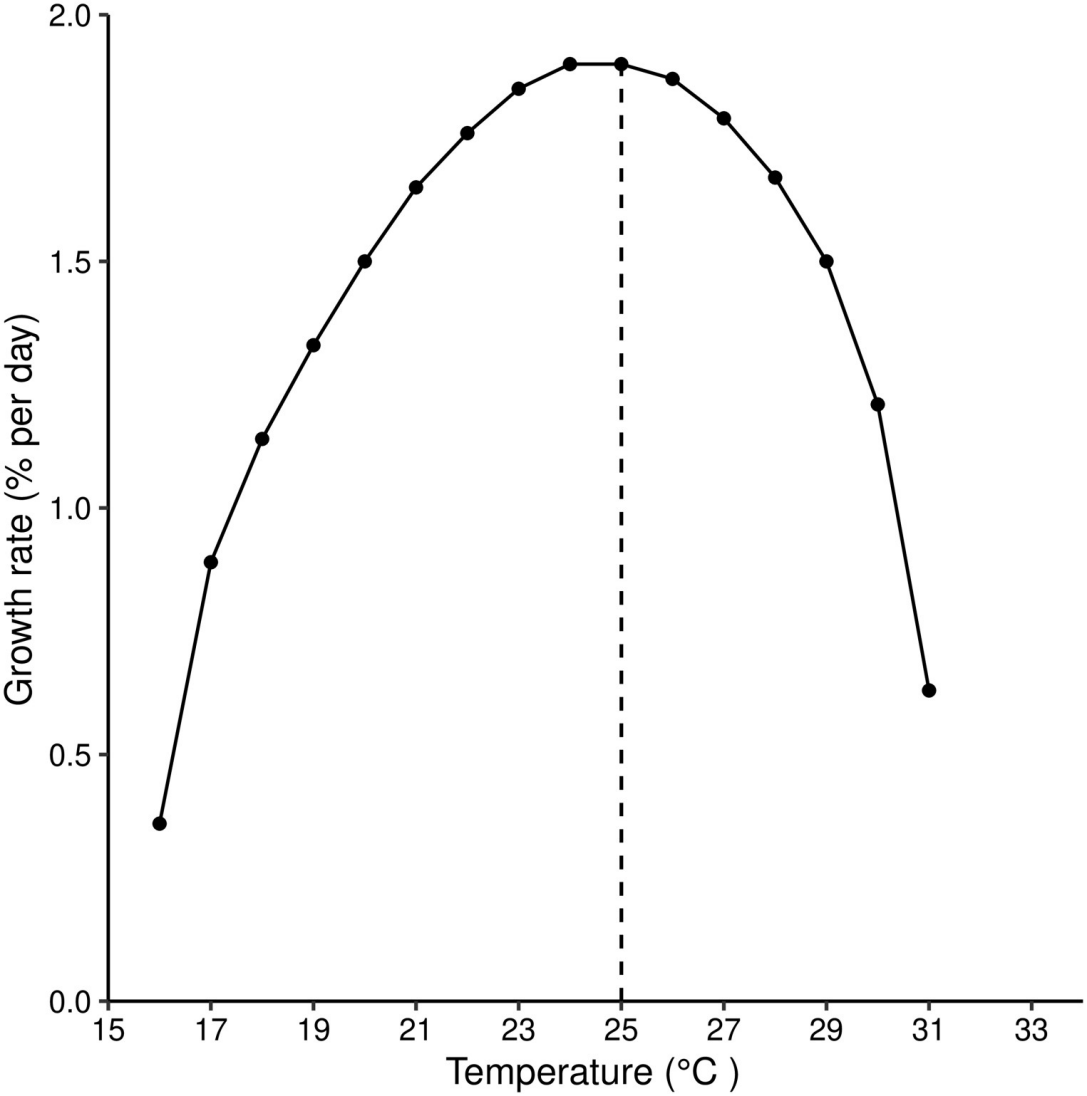
Daily growth rate (%) of a population of *G. m. morsitans* living at different constant temperatures (15°C–35°C). The vertical dotted line at 25°C indicates the temperature at which the growth rate is maximised.

Given that all tsetse species are poikilotherms, which feed on vertebrate blood meals, and which have identical modes of reproduction, it is reasonable to suppose—until demonstrated otherwise—that the mathematical formulae derived above will be qualitatively the same for all species. It is known, however, that the parameters values will vary between species. In the case of *G. m. morsitans* we have some reasonably good estimates for these required parameter values (Table 1). The information is much less complete for all other tsetse species. Strictly speaking, therefore, the results presented below (Figs 1 to 7) refer only to *G. m. morsitans*. Until such time as parameter estimates are provided for other species we will not know how the results differ in these other species.

### Extinction probability as a function of fixed temperatures

Extinction probabilities for *G. m. morsitans* were calculated as a function of fixed temperatures ranging from 15–35°C, for different values of the number of females in the initial population. As expected, sustained extreme temperatures can drive populations to extinction. When the initial population consists of a single female fly, extinction probability did not drop below 0.45 even with optimal temperatures (Fig 3). However, when the starting population consists of even 10 female flies, extinction probability drops rapidly to zero as temperatures increase slightly above 17°C.

With sufficient female tsetse, say 100, in the initial population, extinction probabilities went rapidly to zero when temperatures exceeded 16°C, and remained at 0 even at temperatures slightly above 31°C. No *G. m. morsitans* population, regardless of the number of females in the starting population, can escape extinction outside the range of approximately 16°C–32°C (Fig 3).

### Expected number of daughters per female *G. m. morsitans* for different levels of fixed temperatures

The reproduction number for a population, i.e., the number of surviving daughters an adult fly is expected to produce in her lifetime, is presented in Fig 4 for different levels of fixed temperatures. The reproduction number peaks at 2.8, for temperatures in the neighbourhood of 19°C. It gradually declines as temperatures increase above 21°C, dropping below 1.0 when temperatures exceed about 31°C. However, as temperature decreases below 18°C there is a sharp drop in the reproduction number, which goes below 1.0 at a temperature just below 16°C.

### Time for the population of female tsetse flies to go extinct at different fixed temperatures

The expected number of generations to extinction varies with temperature and with the size of the starting population. When the simulation for the expected number of generations is done for 20 generations, it takes 12 generations for a population starting with 2 female flies to go extinct under optimal temperatures of 19–21°C. As temperature increases above 21°C, the number of generations to extinction reduces gradually, but the decrease becomes much more rapid as temperatures approach 31°C When the number of flies in the initial population increases to 10, extinction did not occur in the first 20 generations for temperatures between 16 and 31°C (Fig 5).

### Growth rates of *G. m. morsitans* populations at different fixed temperatures

Populations of *G. m. morsitans*, which are small enough that we may ignore density dependent effects, grow exponentially for temperature in the approximate range 16–31°C (Fig 6). Notice that, in this figure, growth is plotted as a function of the number of generations completed. The expected number of female tsetse in the population, from the first generation up to the sixth generation, at different levels of temperature, is shown in Fig 6. These results are obtained from $M\left(n\right)=\mu_{T}^{n}$, where *n* is the number of generations completed, and *μ*_*T*_ is the reproduction number. In order to gauge the growth rate as a function of time it is necessary to adjust for the fact that the generation time increases with decreasing temperature.

We define generation time (*γ*) as the expected length of time between the instant a female larva is deposited, and the instant that the resulting adult female deposits the first of her own larvae. That is to say, *γ* = *g* + *ν* + *τ*. The generation time is also temperature dependent—longer during cold seasons and shorter during the hot dry seasons. For example, at 25°C the pupal duration is about 29 days, and the time between adult female emergence and the production of the first larva is about 16 days, giving a generation time of about 45 days. At this constant temperature there would thus be about 365/45 ≈ 8 generation in a year.

To control for generation time, we linearize *M*(*n*) by taking the natural logarithm. Furthermore, we divided ln(*M*(*n*)) by the generation time for different levels of temperature. We plot *M*′(*n*) = ln(*M*(*n*))/*γ* for fixed temperatures from 20°C to 30°C, incrementing by 2°C. The growth rate of tsetse population as a function of fixed temperatures is the slope of *M*′(*n*). After controlling for generation time, the growth rate of the population, as a function of calendar time, is seen to take a maximum value of about 2.0% per day at 25°C, falling away increasingly rapidly towards zero as temperatures approach the upper and lower limits of 32°C and 16°C, respectively (Fig 7).

## Discussion

The aim of this study was to develop a branching process model for tsetse populations experiencing fixed temperatures of different levels, analogous to laboratory situations where tsetse flies are kept under regulated temperatures. We estimate extinction probabilities, times to extinction, expected numbers of female offspring per individual female, and growth rates for each scenario. This enables us to determine temperatures that are optimal for population growth rates, and the lower and upper bound temperatures for the survival of *G. m. morsitans* populations.

Our results confirm findings of earlier studies which suggest that temperature is a key driver of tsetse population dynamics [8, 32, 33]. We show that constant temperatures outside the approximate bounds of 16–31°C are fatal for any population of *G. m. morsitans*. These results are also consistent with observations on populations of *G. pallidipes* in the Zambezi Valley of Zimbabwe, where significant reductions in numbers have been attributed to increasing temperatures, particularly at the hottest times of the year [8].

As temperatures increase, mortality rates increase for adult female tsetse, and for pupae of both sexes [28, 34], but larval production rates also increase and pupal durations decline [17]. In this trade-off, extinction probability initially declines as temperature increase above 15°C (Fig 2). In fact even for quite small pioneer populations the extinction probability falls rapidly to zero for temperatures between 17 and 27°C. Thereafter, however, increases in mortality rates outweigh the increases in birth rates and the extinction probability increases ever more rapidly as temperatures approach 32°C.

When temperatures approach the lower limit of the 16°C– 32°C bracket, adult temperature-dependent mortalities decline to low levels, but female tsetse reproduction rates fall drastically as pupal durations increase. In the extreme, when temperatures drop below 16°C, pupal durations are so long that fat reserves are exhausted before the pupa can emerge [17]. The combination of these factors, if sustained, ensures extinction of *G. m. morsitans* population at these low temperatures (Fig 2). Notice that extreme temperatures affect mature adults less than they affect pupae and newly emerged adults. Thus, at a constant mean temperature of 32°C, mature adults suffer a daily mortality of about 2.8%, which still allows positive population growth as long as the mortality among immature stages is not too high. Mortality among young adults is, however, more than double this level at about 6.1% per day: and, crucially, very few flies survive the pupal stage at 32°C. Similarly, mortality rates among pupae increase exponentially as temperatures decline towards 16°C, whereas loss rates among all adults are smallest at these low temperatures.

Theoretically, for any population to be able to escape extinction, each female adult must produce more than one female offspring, which must themselves survive to reproduce. In epidemiological terms, we then have that the reproduction number, *R*_*o*_ > 1. Fig 4 shows that for temperatures below 16°C, female tsetse will not be able to produce enough female flies to sustain the population. If the cold temperature conditions are prolonged, *Ro* will drop below 1, resulting ultimately in extinction. Thus, as temperatures approach either hot or cold limits, the number of generations that a population can survive goes rapidly to zero even for large pioneer populations (Fig 4).

The reproduction number reached its highest value of 2.8 at 19°C (Fig 4). This may, initially, suggest that the growth rate is highest at that temperature. However, when we calculated the actual growth rate after controlling for the length of generation for different temperature values, we found that the population attains its maximum growth rate at 25°C. This result agrees with published values in the literature [17], where a different method was used to obtain the same results. It also draws attention to the fact that the reproduction number should be used with caution when comparing two populations with differing lengths of generation.

Our findings are in good agreement with experimental, field and modelling studies of the impact of temperature on different tsetse species [8, 32, 35–37], For instance, in [37] an experiment was conducted on three different strains of *G. palpalis gambiensis*, in a bid to determine critical temperature limits for tsetse survival and their resilience to extreme temperatures. For the three strains, a temperature of about 32°C was reported as the upper limit of survival. Our results showed, similarly, that, if the number of female flies in the population is high enough, tsetse population may escape extinction at temperatures slightly above 31°C but will go extinct at higher temperatures. The experiment also reached the conclusion that temperatures of about 24°C are optimal for rearing this species, in good agreement with our modelling results.

As global average temperature continues to rise, Africa has also experienced increasingly high temperatures over the past decades. For instance, in the Zambezi Valley of Zimbabwe the monthly mean temperature has increased by 2°C during the hot dry season [8]. Our results indicate that if the average temperature continues to rise in the Zambezi Valley and other parts of Africa, tsetse populations will go extinct in the hotter parts of the continent, especially in places with similar climate and environmental profile to Zimbabwe. Consequently, future plans for tsetse and trypanosomiasis control and elimination must consider the impact of climate change on tsetse population dynamics in different parts of Africa.

This is a purely theoretical paper. Much more detailed work, beyond the scope of this study, would be required to produce predictions about how growth rates of particular populations would be affected by various projected changes in temperature profiles. Moreover, deciding on the likelihood of various predicted climate change scenarios is again beyond the scope of this study. We are currently working on such problems and, in particular, the question of how to estimate growth rates of populations subjected to the real-life situations where temperatures cycle daily and annually.

## Limitations of the study

Extinction probabilities for tsetse populations have not previously been estimated as a function of temperature. Our modelling framework took into consideration the fact that, as poikilotherms, tsetse mortalities, and rates of larval deposition and pupal development are all temperature dependent. However, the present study did not consider field situations where temperatures vary continuously with time. A modelling framework which will consider this more realistic situation is under construction.

In this study, we considered closed tsetse populations, where there was no in-or-out migration. Estimation of extinction probabilities for populations that are open to migration are markedly more complex and were beyond the scope of the present study. We do note, however, that a preliminary study found that if tsetse populations are in patches, which can compensate for each other, then extinction probabilities are lower than in closed population models [38]. Further work in this area is called for.

We caution that the modelling here is restricted to situations where population numbers lie below the level at which density dependent effects play a significant role. When numbers are larger, however, suppose pupal and/or adult mortality increases with density. Then the temperature-dependent mortalities quoted here provide a lower limit of the true mortality at that time, for any given temperature. Similarly, if density dependence results in a decrease in the birth rate, then the birth rates quoted here provide an upper bound to the true birth rate. For such a population, it then follows that the growth rate at any temperature will be lower than calculated here. Moreover, as temperatures increase, or decrease, towards the upper or lower bounds, respectively, for positive population growth, the population will decline rapidly towards levels where the density-dependent effects fall away. At that point the further dynamics of population growth would be subject to the vital rates used in this study.

Temperature is, of course, only one of many factors that decide whether or not tsetse will occur at all in an area and, if they do, the rate at which their populations may be expected to grow. Other climatic, and vegetation, factors, such as rainfall and normalized difference vegetation index (NDVI), have been shown to be good indicators of the presence or absence of tsetse [39]. Tsetse will not occur, however, in areas where there are no hosts—regardless of the climatic suitability for the flies [40]. Similarly, tsetse populations often disappear from areas that are climatically suitable, through the effects of human activities [41]. Given these levels of complexity, the detailed analysis required to judge the circumstances under which individual, real, tsetse populations would go extinct, is beyond the scope of this paper. What we have done is to provide the theoretical framework for workers, with access to the climatic, biological, and other data, to be able to predict the effects of temperature changes, when viewed in the context of these other variables.

In closing we repeat the caution that the quantitative results shown in Figs 1–7 apply to *G. m. morsitans*. Further laboratory and field work would be required in order to generate many of the input parameters required for other species.
